# From Stein-Leventhal to PMOS: Is It Time to Move Beyond the Ovary?

**DOI:** 10.5812/ijem-175499

**Published:** 2026-11-01

**Authors:** Fahimeh Ramezani Tehrani, Samira Behboudi-Gandevani, Fereidoun Azizi

**Affiliations:** 1 Reproductive Endocrinology Research Center, Research Institute for Endocrine Molecular Biology, Research Institute for Endocrine Sciences, Shahid Beheshti University of Medical Sciences Tehran Iran; 2 Foundation for Research & Education Excellence Vestavia Hills, Al USA; 3 Faculty of Nursing and Health Sciences, Nord University BodØ Norway; 4 Endocrine Research Center, Research Institute for Endocrine Disorders, Research Institute for Endocrine Sciences, Shahid Beheshti University of Medical Sciences Tehran Iran

**Keywords:** Polycystic Ovary Syndrome, Polyendocrine Metabolic Ovarian Syndrome, Nomenclature, Metabolic Dysfunction

The syndrome first described by Irving F. Stein Sr. and Michael L. Leventhal in 1935 has undergone a major conceptual transformation in modern medicine. What began as a gynecologic condition defined by enlarged polycystic ovaries, menstrual dysfunction, and infertility is now recognized as a complex systemic disorder involving metabolism, endocrine regulation, reproduction, and long-term health. Over the past nine decades, accumulating scientific evidence has progressively shifted the paradigm from characterizing the ovary as the primary locus of disease to recognizing a systemic metabolic etiology.

The recent international consensus to rename polycystic ovary syndrome (PCOS) as polyendocrine metabolic ovarian syndrome (PMOS) (1), following a global, multistep process involving over 22,000 patients, clinicians, and researchers, reflects this progress. By incorporating the terms polyendocrine and metabolic into the name, PMOS acknowledges what decades of research have demonstrated: metabolic dysfunction is not a secondary feature of the syndrome but a central feature of its pathophysiology (2, 3). Although this proposal marks an important conceptual advancement, the terminology remains firmly bound to the ovary. The continued retention of an ovarian label merits direct scrutiny because it paradoxically perpetuates the organ-centric perspective that the renaming initiative sought to transcend. Stein and Leventhal named what they could observe: Enlarged polycystic ovaries in women who experienced amenorrhea, infertility, and hirsutism. For decades, the ovary remained conceptually central, and diagnosis revolved around its appearance. The advent of transvaginal ultrasound revealed the fundamental limitation of this framework: polycystic ovarian morphology is present in up to 25% of healthy women and absent in a substantial proportion of those meeting other diagnostic criteria (4). The morphology did not define the disorder.

What replaced it was more compelling. Evidence accumulated over subsequent decades demonstrated that insulin resistance, hyperinsulinemia, dyslipidemia, altered adipose tissue function, chronic low-grade inflammation, and an increased risk of type 2 diabetes and cardiovascular disease are fundamental components of the syndrome (2, 5-11). These metabolic features are present even in many individuals who are not obese and often appear before reproductive symptoms become clinically evident. Studies further demonstrated that insulin acts directly on ovarian theca cells to enhance androgen production, providing a mechanistic link between metabolism and reproductive dysfunction (12). Rather than existing as separate processes, metabolic and reproductive abnormalities are closely interconnected.

More recently, advances in genetics, developmental biology, and systems medicine have further shifted our understanding. Genome-wide association studies have identified susceptibility loci involving metabolic regulation, neuroendocrine function, gonadotropin secretion, and steroidogenesis, rather than exclusively ovarian pathways (13). Evidence from animal models links prenatal androgen exposure and altered metabolic programming to the development of this syndrome in female offspring, as well as to metabolic consequences in offspring of both sexes (14-16). These findings suggest that, under this reconceptualization, the ovary is a target organ—a site at which systemic biological dysregulation becomes clinically visible—rather than the origin of the disorder.

PMOS captures an important truth: metabolism deserves recognition alongside reproduction. Yet it also raises an important question: If the purpose of changing the name is to better reflect current scientific knowledge, should the ovary remain central to that name?

Increasing evidence indicates that the biological processes underlying PCOS are not limited to females (17). During the past decade, studies have consistently reported elevated rates of insulin resistance, adverse body composition, dyslipidemia, hyperandrogenism, and early-onset androgenetic alopecia in brothers and fathers of affected women compared with controls (18-22). Hormonal profiling in these men has identified altered gonadotropin pulsatility, elevated dehydroepiandrosterone sulfate, and abnormal adrenal androgen responses that parallel the neuroendocrine profile observed in their female relatives (23). Some investigators have proposed that early-onset androgenetic alopecia combined with hyperinsulinemia represents the male phenotypic expression of the same underlying biological predisposition (23).

This evidence is preliminary and heterogeneous. Diagnostic criteria for a male equivalent do not exist, sample sizes have generally been modest, and most studies rely on family-based designs rather than population-representative cohorts. However, the consistency of these observations suggests that the syndrome may represent a shared inherited biological condition with sex-specific clinical manifestations. Women express the disorder through ovarian dysfunction and reproductive symptoms, whereas men exhibit predominantly metabolic and endocrine features. This pattern is consistent with that of many genetic diseases in which the same biological mechanisms produce different phenotypes depending on sex.

Retaining “ovarian” in the new name therefore presents a conceptual problem. If PCOS/PMOS represents a heritable polyendocrine metabolic condition, as the new name claims, and if males who carry the same genetic predispositions manifest metabolic and endocrine features consistent with the disorder, then anchoring the condition’s name to a female reproductive organ creates a structural exclusion that may delay recognition, impede research, and prevent affected men from receiving appropriate evaluation or treatment. The history of PCOS is, in part, a history of the consequences of misframing: naming the condition after its most visible anatomical feature delayed recognition of its systemic biology for decades. Retaining “ovarian” risks repeating the same pattern. The term “ovarian” in PMOS carries two implicit claims: that the condition is fundamentally female and that ovarian dysfunction is its defining feature. Both claims are increasingly difficult to sustain. The syndrome involves hypothalamic-pituitary neuroendocrine regulation, adrenal androgen secretion, hepatic insulin signaling, adipose tissue biology, pancreatic beta-cell function, and vascular physiology—a pattern of dysregulation in which the ovary is one affected organ among many. Framing the condition around the ovary reflects a reductionist view that the rest of the new name explicitly rejects.

If the purpose of renaming was to align terminology with current biological understanding, the transition from polycystic ovary syndrome to polyendocrine metabolic ovarian syndrome was an important step forward, but perhaps not the final one. The remaining question is whether “ovarian” should continue to define a disorder that is increasingly understood as systemic rather than organ-specific.

One possible alternative would be to replace “ovarian” with “reproductive.” A term such as polyendocrine metabolic reproductive syndrome would preserve reproductive dysfunction as a defining clinical feature while avoiding restriction to a single organ. It would also remain conceptually compatible with the growing evidence that the syndrome may have sex-specific manifestations. Women primarily present with reproductive dysfunction and hyperandrogenism, whereas men appear to exhibit predominantly metabolic and endocrine features. Although diagnostic criteria for a male phenotype have not yet been established, accumulating evidence raises the question of whether these manifestations represent different expressions of the same underlying biological disorder. The reproductive system encompasses ovarian function as well as hypothalamic-pituitary regulation, gonadotropin secretion, fertility, and reproductive endocrinology, providing a broader and more biologically inclusive framework than the ovary alone.

This proposal is not without limitations. In clinical practice, the term reproductive remains strongly associated with female reproductive health, and the proposed male phenotype appears to be characterized primarily by metabolic and endocrine abnormalities rather than reproductive symptoms. It is therefore possible that no currently available terminology fully captures the biology of the syndrome. A fully mechanistic, sex-neutral alternative might ultimately prove more accurate, although it would sacrifice the clinical familiarity preserved by reproductive and ovarian terminology. A definitive answer may not yet be possible given current evidence. However, acknowledging this uncertainty should not prevent discussion of whether ovarian remains the most appropriate descriptor for a disorder whose underlying mechanisms extend beyond a single organ and potentially beyond a single sex.

The immediate priority is the successful adoption and implementation of PMOS, which represents genuine scientific progress and should not be undermined by another premature revision. This adoption should, however, be accompanied by an explicit research agenda: establishing whether a definable biological syndrome exists in males carrying the same genetic predispositions, developing and validating diagnostic criteria, and examining whether early identification improves metabolic outcomes. If this evidence matures, as preliminary data suggest it may, the case for a further nomenclature revision that removes the remaining organ-specific anchor will become compelling.

The history of this field has been one of continuous scientific refinement. The syndrome was first defined by ovarian morphology, then recognized as a disorder of reproductive endocrinology, and is now understood as a systemic metabolic condition with complex genetic and developmental origins. Emerging evidence for a male phenotype represents the next stage of this evolution. Although the available data are not yet sufficient to justify another immediate change in nomenclature, they raise an important question: can an organ-specific name adequately represent a disorder that may affect both sexes through different clinical manifestations?

Scientific nomenclature should evolve with scientific understanding. The addition of “polyendocrine” and “metabolic” reflects how far the field has progressed since Stein and Leventhal first described the syndrome. As evidence continues to accumulate, the challenge will be to ensure that future terminology reflects the full biology of the disorder, its systemic nature, its genetic complexity, and its potential expression across both sexes. Science has steadily moved beyond the ovary. The name may eventually need to do the same.
